# Actomyosin-Driven Division of a Synthetic Cell

**DOI:** 10.1021/acssynbio.2c00287

**Published:** 2022-09-27

**Authors:** Lucia Baldauf, Lennard van Buren, Federico Fanalista, Gijsje Hendrika Koenderink

**Affiliations:** Department of Bionanoscience, Kavli Institute of Nanoscience Delft, Delft University of Technology, 2629 HZ Delft, The Netherlands

**Keywords:** bottom-up reconstitution, synthetic cell, cell division, actin, myosin

## Abstract

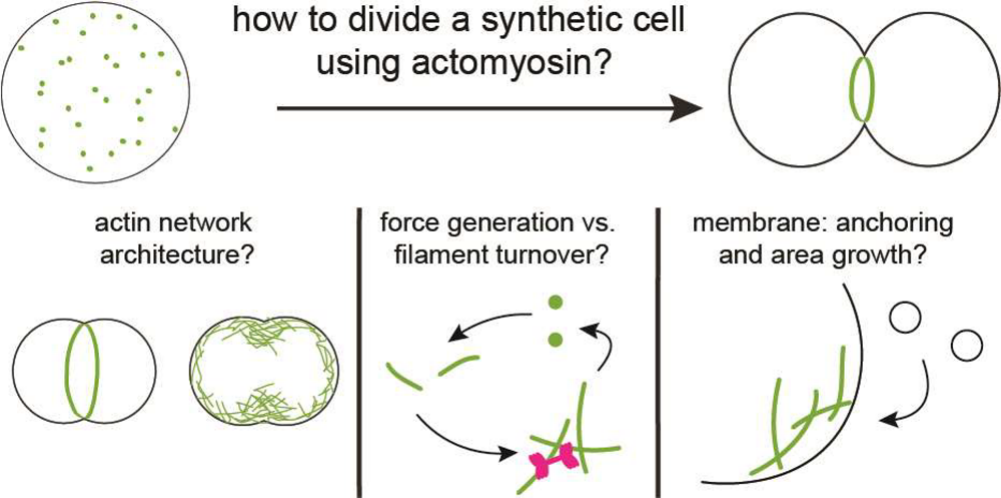

One of the major challenges of bottom-up synthetic biology is rebuilding a minimal cell division machinery. From a reconstitution perspective, the animal cell division apparatus is mechanically the simplest and therefore attractive to rebuild. An actin-based ring produces contractile force to constrict the membrane. By contrast, microbes and plant cells have a cell wall, so division requires concerted membrane constriction and cell wall synthesis. Furthermore, reconstitution of the actin division machinery helps in understanding the physical and molecular mechanisms of cytokinesis in animal cells and thus our own cells. In this review, we describe the state-of-the-art research on reconstitution of minimal actin-mediated cytokinetic machineries. Based on the conceptual requirements that we obtained from the physics of the shape changes involved in cell division, we propose two major routes for building a minimal actin apparatus capable of division. Importantly, we acknowledge both the passive and active roles that the confining lipid membrane can play in synthetic cytokinesis. We conclude this review by identifying the most pressing challenges for future reconstitution work, thereby laying out a roadmap for building a synthetic cell equipped with a minimal actin division machinery.

## 1 Introduction

Bottom-up synthetic biology is an emerging field at the interface of cell biology, chemistry, and physics. Several national and international initiatives have been founded recently, which are aimed at reconstituting synthetic cells that can autonomously grow and divide.^1,2^ As a chassis, usually giant unilamellar vesicles (GUVs) are used, which are cell-sized (5–50 μm) containers enveloped in a lipid bilayer.^3−6^ One of the key functions that a synthetic cell must be able to perform in order to be considered lifelike is cytokinesis,^7^ a process in which a cell physically splits into two daughter cells. To reconstitute cytokinesis, various strategies are being pursued, inspired by biological strategies employed by prokaryotic, archaeal, or eukaryotic cells.^7,8^ These biological systems have in common that cell division is accomplished by a cytoskeletal protein machinery, often ring-shaped, that assembles at the cell equator. In microbial cells (bacteria and yeast), which have a cell wall, this protein machinery has to collaborate with a complex cell wall synthesis machinery.^9,10^ By contrast, animal cells lack a cell wall, and cytokinesis is entirely driven by the actin cytoskeleton. Actin-based cell division could thus be an ideal basis for engineering synthetic cell division.

Bottom-up reconstitution of actin-based cell division is interesting not only from an engineering perspective but also as a means to understand how cytokinesis works at the molecular level in animal cells. Although cytokinesis is a well-studied cellular process, surprisingly many fundamental questions about its working principles remain unanswered:^11^ What are the relative roles of molecular motors and other components and cellular processes in force generation? How much molecular complexity is needed to ensure that the actin cortex retains its structural integrity during cytokinesis? What are the requirements for cortex–membrane interactions to promote furrow ingression? These questions are difficult to address in cell-based studies given the enormous molecular complexity of cells combined with inbuilt redundancies and substantial variation between cytokinetic mechanisms employed by different cell types and organisms.^10,12,13^

In this review, we propose a roadmap toward the bottom-up reconstitution of actin-driven cytokinesis in minimal cells. For brevity, we consider only the process of furrow ingression, neglecting other aspects such as membrane abscission and chromosome and cytoplasmic segregation, which are reviewed elsewhere.^14−17^ Based on theoretical models of cytokinesis in animal cells, we first identify four central biophysical requirements for actin-driven furrow ingression. Next we review experimental insights obtained from recent efforts to reconstitute minimal actin systems. We also emphasize the importance of controlling the surface area of the synthetic plasma membrane to enable cell division. Finally, we propose a roadmap toward building a molecular machinery that can successfully deform a minimal cell-like container.

## 2 Biophysical Requirements for Making a Cell Divide

**Figure 1:**
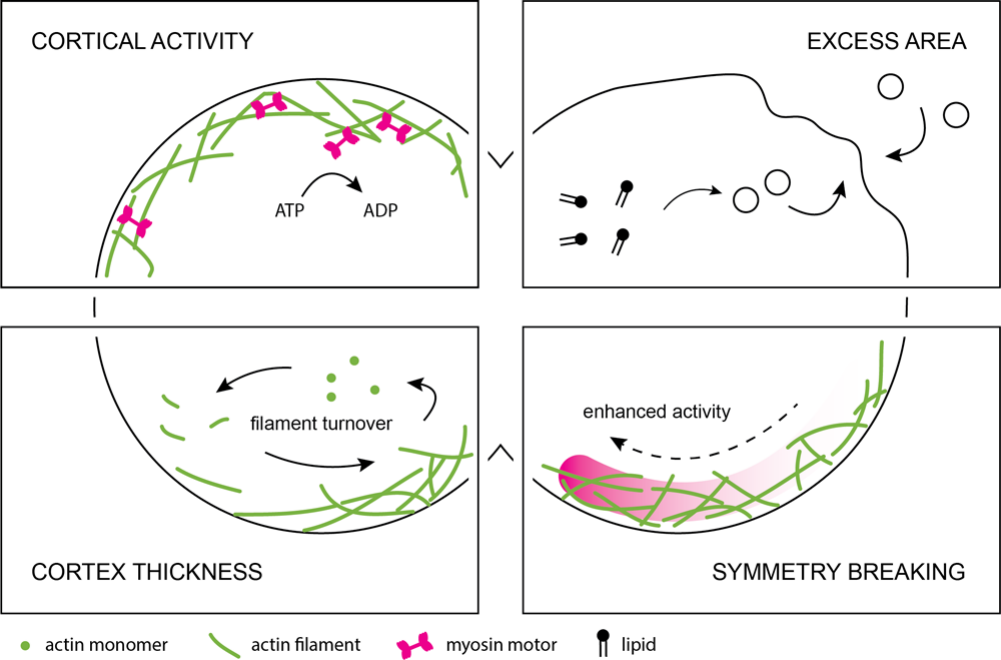
Four key biophysical requirements for reconstituting synthetic cell division. For cell deformation to occur, cortical activity driven by ATP hydrolysis is required (top left), which can for example be generated by myosin activity. Regulation of the cortex thickness (bottom left) is essential for control of cortical activity and is determined by the rate of actin filament turnover versus cortical flows. For cortical activity to lead to cell deformation, the symmetry of the system needs to be broken (bottom right). Finally, to accommodate the drastic change in surface-to-volume ratio during cell division, excess membrane area needs to be generated prior to or during cytokinesis (top right).

Cytokinesis in animal cells is a complicated process that involves many different molecular components (lipids and proteins) whose interactions and localization are tightly regulated. At a coarse-grained level, however, it is possible to formulate general biophysical requirements for cell division based on a consideration of the mechanical forces at play. Pioneering experimental work from the 1950s onward has demonstrated that cytokinesis is accompanied by membrane furrowing,^18^ cortical stiffening,^19,20^ and the appearance of ordered filamentous structures in the cytokinetic ring.^21,22^ These observations have served as input for coarse-grained theoretical and computational models that describe cytokinesis as the shape evolution of a thin, viscoelastic, and active shell around a (nearly) constant volume of cytoplasm. From the models, we can infer several key requirements that a cell, living or synthetic, must fulfill in order to successfully divide (Figure 1):

**1. Cortical Activity.** The actin cortex driving cytokinesis in animal cells must be active. This means that it should include elements that hydrolyze adenosine triphosphate (ATP), an energy-carrying nucleotide, to generate contractile forces that produce cellular shape changes. The viscoelastic and active nature of the cortex can be described using the framework of active gel theory as proposed by Kruse *et al.*(23) This formalism is typically applied in the viscous limit^24−27^ because cytokinesis is slow (minutes) compared to the fluidization time scale of the actin cortex (10 s).^24^ This effectively implies that the cortex flows on the time scale of cytokinesis, which can result from different microscopic origins such as cross-linker or filament turnover.^28,29^ The molecular origins of active force production are complex and depend on molecular detail, as discussed below.

**2. Cortical Thickness.** Active gel theory predicts that cortex activity, at least when mediated by myosin motors, is roughly proportional to cortical thickness.^24−26^ To maintain cortical activity, the cortex must consequently be of a controlled thickness. Cortical thickness is regulated by a balance of actin polymerization and depolymerization (or turnover) and cortical flows: cortical flows accumulate material in the cytokinetic furrow, whereas turnover redistributes actin throughout the cell. This suggests two requirements for synthetic cell division. First, components of the cortex must be laterally mobile to be effectively redistributed by cortical flows.^24,26,30^ Second, actin turnover rates must be low enough to allow local actin accumulation and therefore increased contractility in the furrow region. If actin is removed too rapidly, furrow constriction slows down significantly and may be halted altogether.^26^ On the other hand, complete lack of filament turnover in a 2D actomyosin cortex is theoretically predicted to lead to irreversible clustering of actin, inhibiting effective stress generation.^31^ While active gel theory has been useful to capture various aspects of the actin cortex, recent studies show that other models might be required to describe the mitotic region, where cortex thinning due to protein alignment is accompanied by increased rather than decreased cortical tension.^32^ Interestingly, experimental evidence suggests that in yeast cells the persistent presence of filamentous actin, rather than turnover, is key for successful contraction of the cytokinetic ring.^33^ This difference might be explained by the fact that in yeast the ring is an isolated one-dimensional object, for which theoretical models predict sustained contraction at both slow and rapid turnover.^34^

**3. Cortical Symmetry Breaking.** From the 1930s onward, various models have been proposed to explain the mechanical basis of cytokinesis. The early models range from active expansion of the cell poles^35^ through active pushing by the mitotic spindle^36^ to spindle-mediated relaxation of the cell poles^30,37^ and finally to active constriction of the cytokinetic furrow.^22,26,38,39^ While details vary widely between these models, they share a key characteristic: they all posit that there must be a difference in activity between the polar and equatorial regions to drive furrow ingression. After decades of research, it is now widely accepted (reviewed in ref (40)) that the main driving factor of animal cell cytokinesis is actin-based constriction at the cleavage furrow. However, *in vitro* reconstitution may be the ideal tool to understand actin’s role in molecular detail and to assess the extent to which other mechanisms, such as polar expansion^41^ or interaction with astral microtubules (reviewed in ref (42)), also contribute.

**4. Regulation of Cell Surface Area and Volume.** Consistent with observations in cells,^43^ models have generally assumed that the cytoplasm is very weakly compressible or noncompressible.^26,30^ The apparent cell surface-to-volume ratio, however, changes dramatically during cytokinesis.^44^ It follows that the cell’s (visible) surface area must change. In theoretical works this change in surface area is generally assumed to be energetically “free”, as living cells can regulate the available membrane area through a variety of processes like blebbing^45^ or disassembly of caveolae and membrane trafficking.^46,47^ This supply of membrane on demand is probably one of the most challenging aspects to recapitulate in a reconstituted system.

## 3 Roadmap toward Actin-Driven Synthetic Cell Division

**Figure 2:**
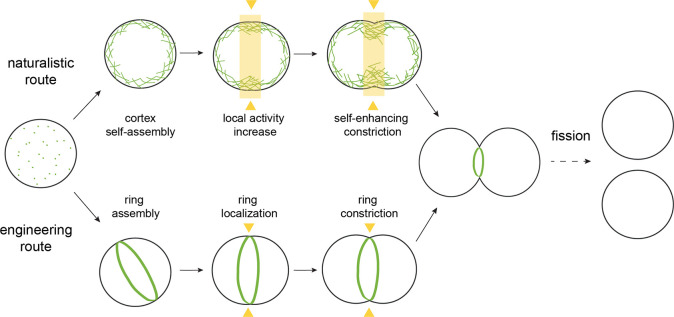
Routes to actin-based synthetic cell division. There are two main routes to achieve actin-driven division of a synthetic cell: by symmetry breaking of a reconstituted actin cortex, triggered by external or biochemical cues, which leads to self-enhanced furrow constriction (the “naturalistic” route, top), or by construction of a contractile ring at the cell equator (the “engineering” route, bottom). Yellow arrowheads indicate where contractile activity is concentrated. The final fission step is outside the scope of this review.

Cytokinesis of animal cells is a highly complex and tightly regulated process. Nevertheless, as discussed earlier, fairly minimal computational models are able to recapitulate aspects of cytokinesis, suggesting that the underlying mechanisms may be recreated with simplified molecular mechanisms. Here we propose a roadmap toward reconstituting actin-driven cell division by considering lessons from recent cell and *in vitro* (*i.e.*, cell-free reconstitution) studies. Basically, there are two routes for reconstitution of actin-driven cytokinesis (see Figure 2). First, cell division can be recreated *via* reconstitution of an actin cortex that, upon symmetry breaking, is more contractile at the cell equator than at the poles. This route is closest to cytokinesis in mammalian cells, and we therefore name it the naturalistic route. The second route is by construction of a cytokinetic ring that anchors and contracts at the cell equator, coined the engineering route. We will first discuss the design of an actin-based machinery fit for driving cytokinesis in both scenarios, and in the next section we will consider the design of the lipid membrane envelope.

### 3.1 Naturalistic Route: Building a Self-Assembling Cytokinetic Ring

**Figure 3:**
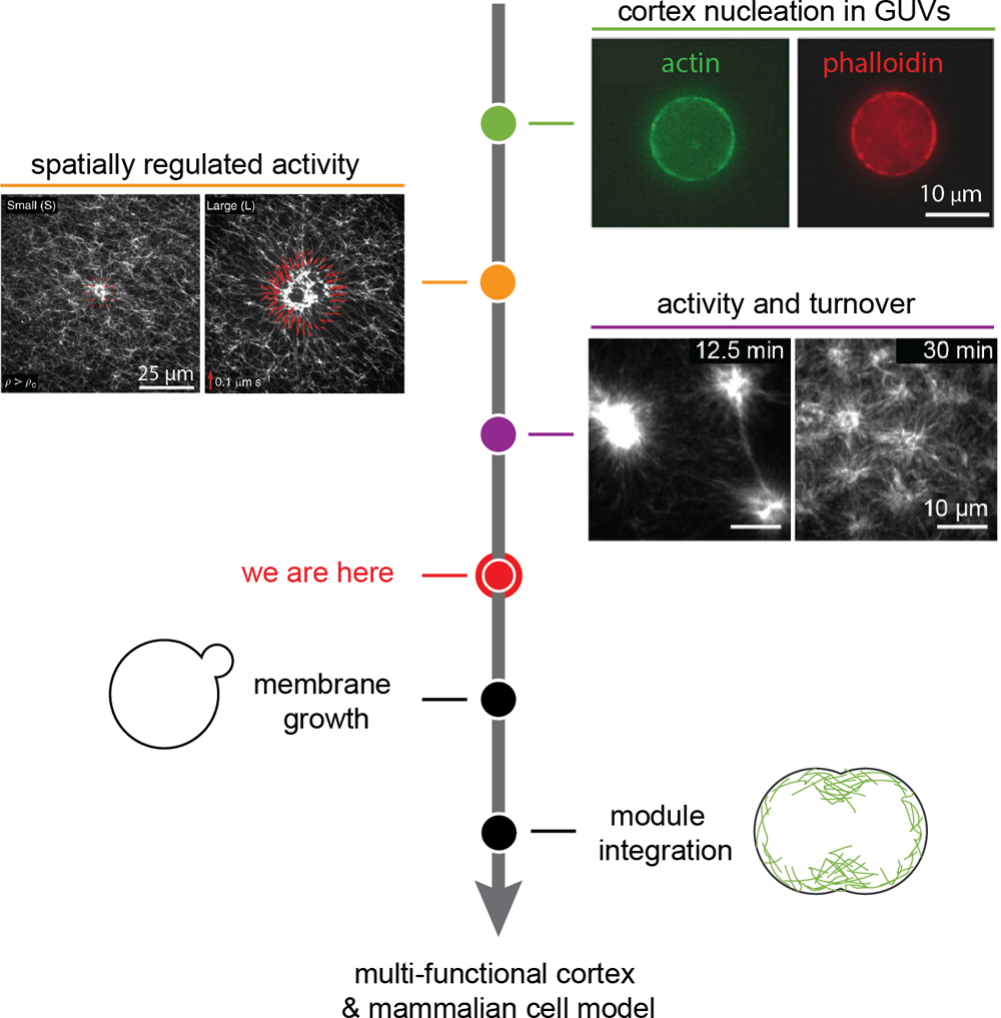
Roadmap to division with an actin cortex. Green: successful nucleation of an actin cortex inside GUVs (reproduced with permission from ref (67), copyright 2009 Biophysical Society). Filament formation of actin (green) is confirmed by colocalization of the filament-binding peptide phalloidin (red). Orange: spatiotemporal control of myosin activity by light-induced inactivation of the myosin inhibitor blebbistatin was used to generate network contraction over different length scales, from small (left) to large (right) (from ref (68), CC BY 4.0). Purple: combination of myosin activity with actin filament turnover generates sustained network contraction (from ref (63), CC BY 4.0). In the coming time, steps need to be taken to engineer membrane growth and finally to integrate the different modules inside a GUV.

During interphase, mammalian cells have a continuous actin cortex that lines the plasma membrane.^48^ When cells enter mitosis, the cortex is remodeled and self-assembles into a contractile ring at the cell equator. Symmetry breaking and midplane localization of the cytokinetic furrow are initiated by biochemical signaling, which includes Rho-dependent myosin phosphorylation in the furrow region.^26,49^ The locally enhanced activation of myosin is thought to lead to cortical flows from the poles to the equator,^30,50^ which further accumulate and organize contractile elements in the furrow^51^ that drive furrow ingression.^26^ Such a complex self-assembling system has not been built to date, but steps have been taken along the road (Figure 3).

#### 3.1.1 Reconstitution of Active Actin Networks

Both cell-free experiments and theoretical models of cortex-like disordered actin networks have been used to elucidate why disordered actomyosin networks are contractile in the first place. The detailed mechanisms have been reviewed elsewhere,^52−54^ but they broadly comprise two scenarios. Actin filaments are semiflexible polymers with a thermal persistence length of 10–15 μm, which is on the same order as their typical contour length in *in vitro* studies.^55^ It should be noted that cortical actin filaments are much shorter *in vivo*, ranging from 120 to 1200 nm depending on the nucleator and cell type.^56^ The first contraction scenario, which is relevant for well-connected networks of long filaments, is that the anisotropic mechanical force–extension response of actin filaments causes them to buckle and break under motor-induced compressive stress.^57,58^ The second scenario, which is relevant for networks with short actin filaments, is that the structural polarity of actin filaments in combination with the tendency of myosin II motors to dwell at the filament plus end before detachment causes contraction *via* polarity sorting.^25,59,60^ In the actin cortex of mammalian cells there may be a combination of both mechanisms since distinct populations of short and long filaments are present there.^56^

Notably, the combined effect of contractile motor activity and actin turnover remains poorly explored. Theoretical models generally assume that the cytokinetic cortex does undergo actin turnover^24,26,61^ and have even indicated that turnover is key for sustained stress generation during furrow ingression.^31^ Experimentally, besides one study with a cell extract,^62^ only one minimal *in vitro* study to date has combined actin turnover and myosin activity.^63^ That work showed that myosin activity alone can be sufficient to induce turnover in minimal actin networks (see Figure 3, purple). Myosin-driven compaction and fragmentation of Arp2/3-nucleated actin led to the removal of actin from the network and subsequent redistribution and reincorporation of network components, creating a cortex in a dynamic steady state. Strikingly, actin turnover rates were observed to be much lower here than typical rates in cells, with actin turning over within tens of minutes rather than tens of seconds.^64,65^ This discrepancy is likely due to the absence of dedicated actin-severing proteins in the minimal system. More rapid turnover has been observed *in vitro* in volume-spanning entangled actin networks where filaments were severed by cofilin and polymerization was driven by formin.^29^ Combining more rapid turnover with motor activity *in vitro* may open a rich field of network behaviors, with complex implications for the regulation of both cortical thickness and stress propagation and relaxation.^66^

To build and control a system that allows actin to turn over, we can turn to the growing body of work studying the functions of various actin regulators on the single-molecule or filament level. Research into the two key nucleators of cortical actin, Arp2/3 and formins,^56,69,70^ has uncovered new complexities in recent years. Both the processivity and the actin filament elongation rate of different formins have been shown to be regulated by the physicochemical environment, the presence of profilin, and mechanical stress.^71−73^ Even more complex coregulation of formin with other barbed-end binding proteins is emerging.^74^ Regulation of Arp2/3 by profilin^75,76^ as well as by actin filament curvature^77^ has been known for a number of years. However, the true diversity and complexity of the various isoforms of Arp2/3, which itself is a protein complex consisting of seven protein subunits, is only just emerging.^78^ In addition, there are regulating factors that control cortex architecture by modulating both formin and Arp2/3 activity.^70^ Besides formin and Arp2/3, other actin nucleators such as the recently identified spire^79^ have barely been used in reconstitution experiments and may offer yet other routes toward reconstituting a minimal dynamic cortex. Actin depolymerization can equally be controlled by various factors. Disassembly of filamentous actin *in vitro* is usually mediated by proteins of the ADF/cofilin family.^80^ The activity of ADF/cofilin proteins has been shown to depend on cooperation with other proteins^81,82^ and on actin cross-linking.^83^ ADF/cofilin also facilitates debranching in actin networks nucleated by Arp2/3,^80^ which is furthermore sensitive to force and actin filament age.^84^

#### 3.1.2 Reconstitution of Actin Cortices Inside GUVs

Controlled actin encapsulation in GUVs has proven to be a challenge. Over the years, many different methods have been explored for protein encapsulation, based on either lipid swelling^85^ or emulsion transfer^67,86−88^ (reviewed in ref (3)). Of these, methods based on emulsion transfer are currently the most successful, although the encapsulation efficiency and the ability to scale-up the number of encapsulates remain to be characterized.^87^ Most prior GUV studies focused on the effect of cross-link proteins and myosin motors on bulk-nucleated actin. By contrast, membrane-nucleated actin networks with turnover in GUVs remain poorly explored. Early works from the Sykes lab^67,89^ demonstrated that Arp2/3-nucleated cortices can be reconstituted at the inner leaflet of GUVs (Figure 3, green) and that such cortex-bearing vesicles reproduce aspects of the mechanics of living cells. More recently, Dürre *et al.* demonstrated that Arp2/3-nucleated cortices can induce local deformations of the GUV membrane by either polymerization forces alone or in combination with contractility induced by nonmuscle myosin-II.^90^ New work from the Liu lab shows that membrane-bound Arp2/3 in combination with fascin and α-actinin is sufficient to yield ringlike membrane-bound actin networks.^91^ Myosin-initiated contraction of these networks resulted in membrane constriction, thus getting one step closer to cell division.

More extensive work, especially with myosin-driven cortices, has been performed with stable actin filaments anchored to the membrane by streptavidin- or actin-binding membrane proteins. In such systems, cortical tension was shown to depend on the ratio of active versus passive cross-linkers,^92^ and excessive cortical tension was shown to cause full or partial detachment of the cortex from the membrane.^92,93^ Recently, Litschel *et al.* demonstrated the formation of actomyosin rings in GUVs.^86^ However, these structures were unable to deform the GUV membrane on large length scales because they slipped on the membrane. Based on our understanding of cell division, this is likely due to (at least) three missing factors: cortex turnover, symmetry breaking between the poles and equator of the synthetic cell, and a severely limited supply of extra membrane area. Symmetry breaking is likely necessary for productive and sustained membrane deformations. There are several artificial means by which symmetry breaking could be triggered in synthetic cells. Myosin activity could, for instance, be locally light-activated by targeting either the light-sensitive myosin inhibitor blebbistatin^68,94,95^ (see Figure 3, orange) or myosin-II directly.^96^ Similar approaches could be used to locally modulate the cross-link density of the actin cortex or the interaction strength of the cortex with the synthetic cell membrane. Finally, it would likely help to make GUVs shape-asymmetric, for instance by using microfluidic channels.^97^

Conceptually, building a dynamic actin cortex and pushing it toward self-assembly of a cytokinetic furrow is very appealing. Such a system would mimic many core attributes of the cortex of living animal cells. Furthermore, the continuous nature of such a cortex would allow it to take on a triple function: as a mechanoprotective module for the synthetic cell, as a dynamic control of cortical and membrane tension, and as a division apparatus. Its versatility sets the actin cortex apart from other cytoskeletal systems such as FtsZ.^98^ A lifelike actin cortex offers the opportunity to test existing theoretical models of cell division and to tease out the essential functions needed for cytokinesis in living cells. On the other hand, a dynamic actin cortex will necessarily comprise more proteins and hence a higher level of complexity than one composed of stable actin filaments. From an experimental perspective, reconstituting sustained actin turnover in combination with motor activity in particular will be challenging, as it requires fine control over both stoichiometry and activity of cytoskeletal components. In addition, timing self-assembly in connection with other cellular events, such as chromosome segregation, cell size doubling, and fission, remains a challenge to date.

### 3.2 Engineering Route: Building an Isolated Contractile Ring

A more engineering-type approach to synthetic cell division may also be interesting: instead of building a cortex that self-organizes into a ring, one could build an isolated ring directly (Figure 2, bottom). This would inherently fulfill the requirement for different activities in polar and equatorial contractility, as by definition the poles are not contractile in such a case. If a sufficient supply of long actin filaments throughout furrow ingression can be ensured, the need for controlled turnover may be diminished, and the complexities of such regulated filament assembly and disassembly may be avoidable. This approach will need to address three key challenges: (1) building an actin ring, (2) making it contractile, and (3) maintaining the ring’s midcenter position during contraction such that membrane invagination rather than ring slippage occurs.

#### 3.2.1 Building an Isolated Ring

**Figure 4:**
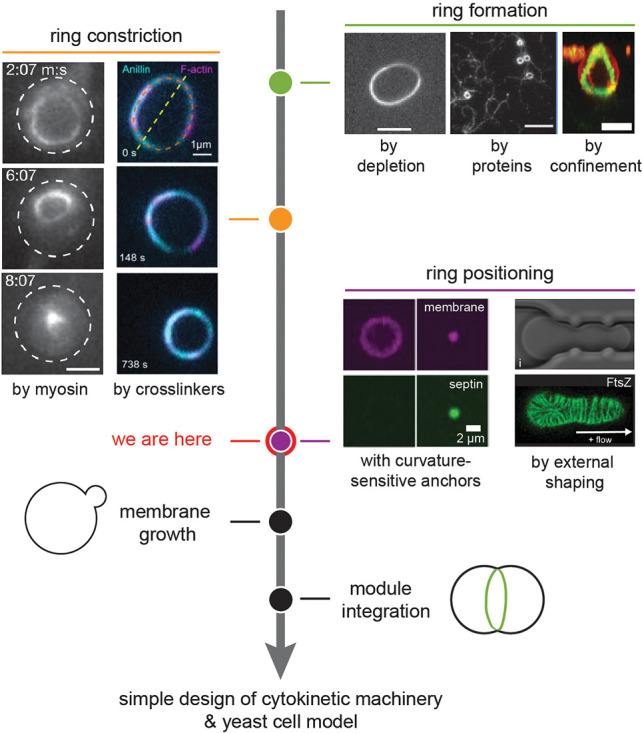
Roadmap toward synthetic cell division using a contractile actin ring. Green: actin rings can be formed by depletion interactions using macromolecular crowders (reproduced with permission from ref (99), copyright 2009 IOP Publishing), by proteins that combine actin binding with curvature generation such as curly (from ref (108), CC BY 4.0), or simply by confinement of actin bundles (reproduced with permission from ref (85), copyright 2015 Royal Society of Chemistry). Orange: constriction of actin rings can be executed using myosin motors (reproduced with permission from ref (110), copyright 2015 Nature Publishing Group) or actin cross-linkers like anillin (from ref (107), CC BY 4.0). Purple: the actin ring can be positioned using curvature-sensing anchors (left: septin binds preferentially to membranes of higher curvatures as shown with membrane-coated beads; from ref (111), CC BY-NC-SA 3.0) or by mechanical deformation (right: microfluidic traps deform GUVs, leading to rearrangement of FtsZ rings; from ref (112), CC BY 4.0). In the next steps toward achieving synthetic cell division, membrane growth needs to be reconstituted, and all separate modules have to be integrated.

Actin filaments can be bundled and bent into ringlike structures in various ways (Figure 4, green). Most simply, ring formation can be induced by entropic effects through macromolecular crowding^99^ or by cross-linking with multivalent ions.^100^ Alternatively, proteins can be used to bend actin into rings. Septins spontaneously bend actin into ringlike structures^101^ and are recruited to the cytokinetic ring, where they cooperate with anillin in actin–membrane binding.^102−106^ Anillin itself also promotes the formation of actin rings by promoting overlap between filaments.^107^ Furthermore, the IQGAP fragment “curly” has recently been shown to bend actin into rings on model membranes by bending single actin filaments.^108^ The fact that all three of these proteins are enriched in the cytokinetic furrow^109^ suggests that these ring-forming capabilities may provide a cellular mechanism to promote successful cytokinesis.

Confinement of actin filaments inside spherical droplets or vesicles tends to promote the formation of actin rings because the confinement forces the semiflexible filaments to minimize the filament bending energy.^113^ Entangled or cross-linked actin networks inside emulsion droplets and inside lipid vesicles form peripheral cortex-like networks,^91,114−118^ while bundled actin forms one or more closed rings.^85,91,110,116,117^ Single rings form when the container size is smaller than the persistence length of the actin filament or bundle.^91,117^ Recent theoretical^119^ and experimental^86^ work has shown that ring formation can be further enhanced by introducing actin–membrane adhesion. It should be noted, however, that ring formation requires a subtle balance of filament–filament and filament–wall adhesion as well as size and stiffness of the confinement and is not trivial to precisely control experimentally.

#### 3.2.2 Making the Isolated Ring Contractile

Contracting a once-formed actin ring can again proceed in different ways (Figure 4, orange). The classical purse-string model posits a well-organized cytokinetic ring that closes by myosin-mediated translocation of actin filaments.^22,120^ Although this model does not appear to hold in all cell types,^121−123^ recent super-resolution and electron microscopy studies showed convincing evidence that it does apply in at least some cell types.^124,125^ Contracting actin–myosin rings have been successfully reconstituted on supported lipid bilayers (SLBs)^108^ and inside water-in-oil droplets^110^ and GUVs.^86^ The efficiency of ring closure is likely determined by the orientation and arrangement of the actin filaments in the ring, which can be tuned by varying the cross-linker composition and concentration.^58,117,126−128^

Alternatively, ring contraction may be driven by mechanisms that do not require molecular motors. For instance, anillin was recently shown to drive actin bundle contraction even though it is a passive cross-linker.^107^ Contraction was attributed to an energetically driven process whereby actin filaments increase their overlap as long as energy can be gained by accumulating diffusive cross-linkers in the overlap region.^107^ This mechanism was enhanced when anillin was combined with actin depolymerization. Since contraction driven by passive cross-linkers does not consume energy from an external energy source such as ATP, it can only bring the system into a configuration of minimal free energy, at which point rearrangement will stop.^129^ Intriguingly, recent theoretical modeling^130^ suggests that a cross-linker that consumes ATP to unbind from actin filaments but does not actively translocate them like myosin could in principle induce contraction indefinitely. In this case, the consumption of an energy carrier breaks detailed balance in the system, and in combination with the asymmetric mechanical properties of actin, overall contractile forces can arise.

#### 3.2.3 Keeping the Isolated Ring in Place

Although contractile actin rings have been successfully reconstituted inside GUVs, to date none of these efforts have yielded anything close to furrow-like membrane invaginations. The rings either detached or slipped along the membrane upon myosin activation,^86,92,93,110^ at best producing rare instances of slight membrane deformation.^86^ In cells, positioning of the cytokinetic ring is ensured by a complex and poorly understood interplay between the actin and microtubule cytoskeleton, local changes in lipid composition, and soluble signaling molecules.^131,132^ Reconstituting this interplay in GUVs seems too technically challenging to be expected in the coming years. We therefore expect that simpler, if less biological, solutions may be more promising. To the best of our knowledge, no such efforts have been reported to date. However, a few options present themselves (Figure 4, purple): curvature-sensing or -inducing scaffolding proteins such as septins^111^ or I-BAR-domain proteins^133,134^ may help in templating a furrow and inhibiting slippage of contractile actin rings. These proteins may have to be combined with more engineering-type solutions designed to deform GUVs from the outside, either by confinement in traps^97,112^ or by membrane-binding complexes.^135−137^

Building an isolated contractile actin ring in principle offers an elegant way to drive synthetic cytokinesis. The formation of such a ring requires only few components, and tuning ring contractility is certainly subtle but most likely achievable. The biggest technical challenge in this approach is to localize the ring at the equator and keep it in place during contraction in order to foster productive membrane deformation. On a more conceptual level, reconstituting isolated contractile rings likely will not bring us much insight into the mechanisms of cytokinesis in animal cells. However, it may be a valid strategy to understand mechanisms in yeast cytokinesis, in tandem with top-down work on yeast cell ghosts.^138^

## 4 Involving the Membrane

So far, we have largely ignored an important assumption in the key requirements that we set out earlier, which is that the GUV membrane and actin cortex are intrinsically coupled. However, it is far from trivial that actomyosin contraction is followed by deformation of the cellular membrane. While actomyosin networks and membranes have separately been thoroughly investigated by biophysicists, their interplay has received much less attention and presents a crucial challenge to address in the coming years.

### 4.1 Membrane–Cortex Anchoring

*In vivo*, a multitude of cytoplasmic proteins are known to be involved in actin–membrane adhesion, many of which have binding sites for both actin and plasma membrane lipids. These proteins include ERM (ezrin, radixin, moesin) proteins, myosin 1b, anillin, and septins.^142−145^ How these proteins cooperate in adhesion and how they are spatially organized at the membrane remains elusive. Electron microscopy and super-resolution microscopy have revealed that the distance between the filamentous actin and the plasma membrane is surprisingly large, ranging from 10 to 20 nm in the cell cortex of animal cells^146^ and from 60 to 160 nm in the cytokinetic ring of fission yeast.^147,148^ It is unclear how this large gap, which is often wider than the distance that known linker proteins span, arises. There is evidence that the actin cortex itself is stratified, with myosin filaments being restricted toward the cytoplasmic side of the cortex due to steric exclusion from the dense cortex.^149^ Interestingly, a recent *in vitro* reconstitution study showed that actin–myosin networks on SLBs spontaneously self-organize into radial actin structures (asters) with myosin at the core and layered atop to relieve steric constraints.^150^

Mechanical measurements on cells indicate that the cortex adheres to the membrane *via* a high density of weak links. With optical tweezers, one can pull membrane tubes from cells with membrane-bound beads. These tubes can easily be moved over the cell surface,^151^ indicating that the membrane easily zips off the cortex and quickly rebinds. Various tube-pulling experiments have shown that the force required for tube extrusion is dependent on the levels of ezrin^152^ and phosphatidylinositol-4,5-bisphosphate (PIP2) lipids.^153^ PIP2 lipids specifically interact with many actin-binding proteins, including ezrin (reviewed in ref (154)). In *Schizosaccharomyces pombe* cells, PIP2 depletion causes sliding of the cytokinetic ring, indicating that PIP2-dependent actin–membrane adhesion is essential for anchoring of the ring.^155^ Although PIP2–protein interactions are individually weak, their high density collectively causes a tight yet dynamic seam between the bilayer and the cytoskeleton.

**Figure 5:**
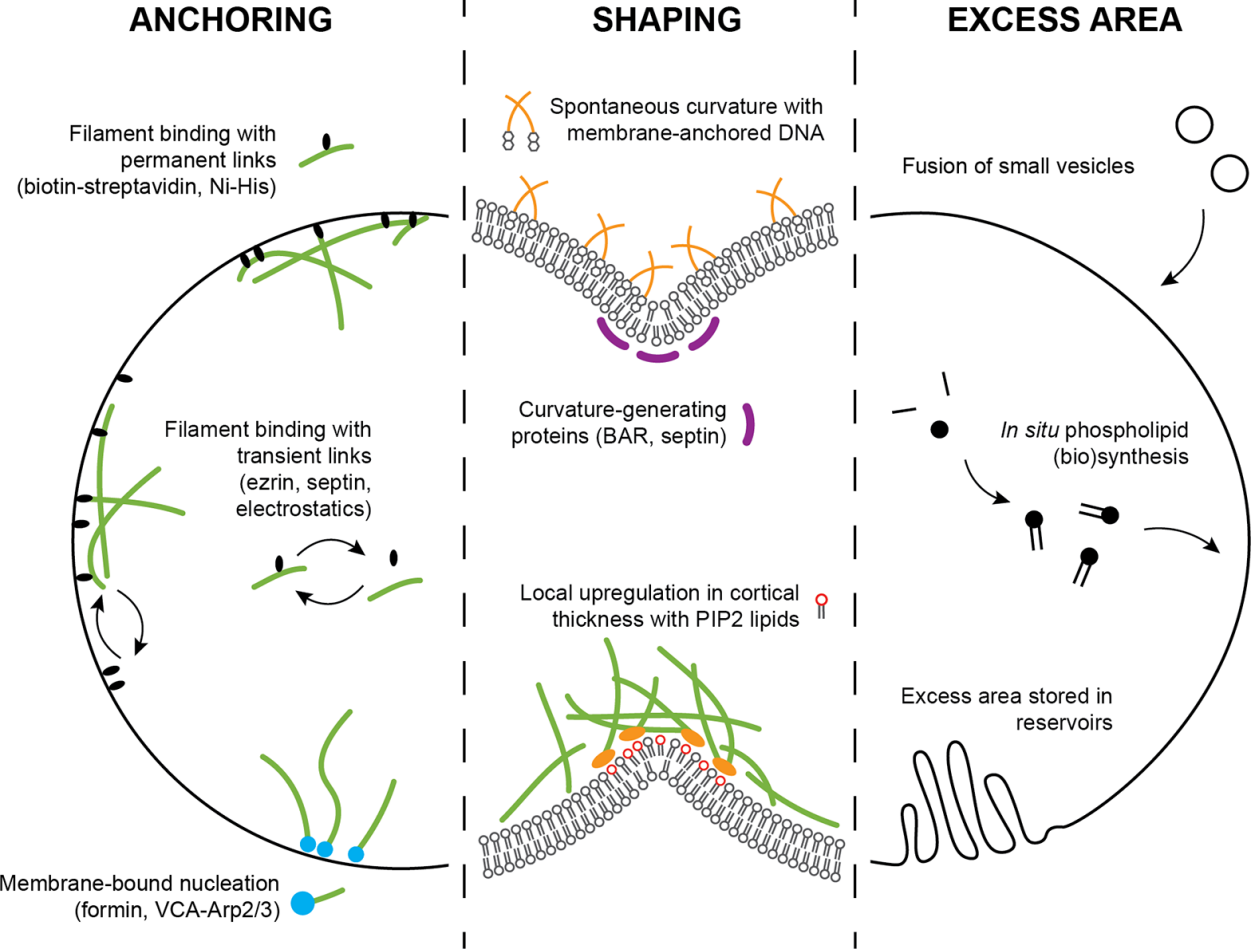
Membrane engineering for synthetic cell division. A schematic overview of possibilities for membrane design is shown. Anchoring of the actin cortex (left) can be done either *via* filament nucleation from the membrane or *via* filament binding to the membrane. Binding can be done using strong permanent linkers or weaker transient links. Membrane shaping (middle) can be done by generating spontaneous curvature, for example with membrane-bound DNA nanostars^139^ or physiological curvature-generating proteins such as BAR proteins^140^ or septin.^141^ Otherwise, when lipids can be spatially separated, local elevation of PIP2 levels can increase cortical thickness *via* regulating actin nucleation and severing proteins. To provide excess area during cytokinesis (right), new membrane area can be added by fusion of small vesicles or by *in situ* synthesis of phospholipids. Alternatively, membrane area could be stored in reservoirs that become accessible upon furrow ingression.

In stark contrast to the reversible actin–membrane binding observed *in vivo*, *in vitro* reconstitution efforts have mostly relied on anchoring interactions with unphysiologically high binding affinity (Figure 5, left). Many studies used either direct coupling of biotinylated actin filaments to biotinylated lipids *via* streptavidin^86,92,156^ or indirect coupling using His-tagged actin-binding proteins coupled to Ni-NTA lipids.^93,157^ These bonds are virtually permanent and unbreakable.^158−160^ Nevertheless, at low anchor densities, actomyosin cortices anchored in this manner still detach from the membrane upon myosin activation,^92,93^ resulting from anchor slippage^161^ or pulling out of lipids.^92^ In two studies with high anchor density, the actomyosin cortex did remain attached to the membrane upon contraction, but it slid toward one side so that the membrane was only minimally deformed.^86,92^ Cortex slippage is likely due to the fluid nature of the lipid bilayer membrane. Actin and microtubule gliding assays with motor proteins anchored onto SLBs have shown that motor activity is accompanied by lipid slippage.^162,163^ The interplay between the dynamics of the actin cortex and the dynamics of the lipids is complicated. Adhesion to the actin cortex slows lipid diffusion,^164,165^ while myosin-driven actin cortex contraction can actively cluster lipids into microdomains.^166−171^ Altogether, it remains poorly understood what conditions are necessary for the actin cortex to remain stably anchored and cause sustained membrane deformation.

Dynamic actin–membrane linkages have to date been reconstituted only on SLBs. Using ezrin recruited to the bilayer *via* PIP2 lipids, a dynamic actin network was created that could be remodeled by passive filament cross-linkers.^172^ Bead tracking microrheology showed that ezrin serves as a dynamic cross-linker for the membrane-attached actin layer, with the network stiffness being controlled by the pinning point density.^173^ Ezrin-anchored actin filaments could diffuse over the membrane, but longer filaments were immobilized, being pinned by a larger number of actin–membrane links.^174^ This indicates that collective binding with transient links can fix cytoskeletal structures in place on top of a fluid membrane. Other promising candidates for *in vitro* transient actin binding are septins and anillin. Septins themselves can bind to membranes and self-assemble into filamentous scaffolds.^175^ Membrane binding is curvature-sensitive,^141,176^ which renders septins interesting candidates for spatial control of actin organization in synthetic cells. In solution, septins can bind and cross-link actin filaments into curved bundles.^101^ This could explain the role of septins in the formation and stabilization of contractile actomyosin rings observed *in vivo*.^101^ However, the simultaneous interplay of septins with lipid membranes and actin has yet to be reconstituted *in vitro*. Like septins, also anillin possesses both actin-binding and membrane-binding domains. Anillin has been shown by reconstitution to be able to anchor actin filaments to lipid membranes in a RhoA-dependent manner.^177^ In combination with anillin’s ability to bundle and constrict actin rings *via* condensation forces,^107^ it would be interesting to explore anillin’s ability to promote synthetic cell division. Besides protein-based binding, actin filaments can also be bound to lipid membranes by electrostatic interactions that can be tuned by the choice of ions, offering an alternative route for studying and modulating transient actin–membrane binding.^178^

Besides actin–membrane linkers, also membrane-localized actin nucleation contributes to cortex–membrane adhesion. The main nucleators of cortical actin filaments *in vivo* are Arp2/3 and formin.^69^ In combination with membrane-bound nucleation promoting factors such as WASP, Arp2/3 is responsible for the formation of branched actin filament arrays, whereas formins nucleate linear filaments. Actin nucleation has been successfully reconstituted *in vitro* both with formins, often for simplicity with constitutively active mutants,^179^ and with Arp2/3, often activated by WASP fragments such as VCA.^67,180,181^ Actin turnover can be introduced by addition of severing proteins such as ADF/cofilin.^182^

It is unknown how filament nucleation in conjunction with actin–membrane anchoring by dynamic linker proteins such as ezrin will influence the ensemble mechanics of the actin–membrane composite. Tailoring actin-based division machineries toward synthetic cell division will require careful tuning of the cortex itself, the anchoring strategy, and also the membrane physicochemical properties.

### 4.2 Membrane Engineering

The membrane should not be considered just a passive player in cytokinesis. In contrast, membrane properties can be exploited to aid cytokinesis, for example by shaping the contractile network (Figure 5, middle). *In vivo*, the plasma membrane in the cleavage furrow has a distinct lipid composition that is thought to contribute to cytokinesis by biochemical signaling and perhaps also by induction of spontaneous curvature.^183^ Elevated PIP2 levels at the cleavage furrow probably contribute to furrow ingression by recruiting anillin, septins, and ERM proteins.^184^ Furthermore, PIP2-mediated signaling promotes the formation and maintenance of a stable actin cortex by promoting actin nucleation and slowing actin filament severing *via* actin regulatory proteins.^185^ Other membrane components such as gangliosides and cholesterol also accumulate in the cleavage furrow, where they regulate and bind the cortex.^184^ In addition, the distribution of phosphatidylethanolamine (PE) lipids over the two bilayer leaflets changes significantly during cell division: while PE lipids reside in the inner leaflet during interphase, they are exposed in the outer leaflet of the cleavage furrow during cytokinesis.^186^ This asymmetric distribution of PE lipids has been shown to be important for the disassembly of the contractile ring after cytokinesis.^186^ It is possible that the specialized lipid composition of the cleavage furrow also directly affects cytokinesis by changing the mechanical properties of the membrane, but this remains to be shown.

For engineering artificial cell division, it could be useful to exploit known mechanical effects of lipids. An important characteristic of lipid bilayers is that asymmetries between the two membrane leaflets give rise to membrane spontaneous curvature. Asymmetries can be generated in many different ways (reviewed in ref (187)), such as by different lipid compositions or different numbers of lipids in the two leaflets,^188^ binding of proteins to one leaflet,^137^ insertion of membrane-anchored DNA oligomers into one leaflet,^139^ or different solutes on the two sides of the membrane.^189^ In the context of actomyosin-based synthetic cell division, spontaneous curvature effects could be exploited for spatial control and symmetry breaking. Binding of proteins to the outer leaflet of vesicles can be used to make vesicles dumbbell-shaped and to constrict and even split the neck.^137^ Generation of negative membrane curvature could be used to locally recruit septins, which selectively bind to membrane areas with micrometric curvature.^111,141^ In addition, membrane-binding proteins that not only sense but also generate curvature could be used, such as BAR-domain proteins.^190^ I-BAR proteins were shown to directly bind to actin in fission yeast^134^ and are therefore interesting candidates for promoting actomyosin-driven membrane invagination. Interestingly, I-BAR domain proteins promote ezrin enrichment in negatively curved membrane protrusions,^133^ providing further prospects for boosting membrane invagination *in vitro*.

### 4.3 Addition of New Membrane Area

To create two daughter cells from a single mother cell, assuming spherical geometry, the cell surface area has to increase by 28%.^26,44^*In vivo*, this extra membrane area is delivered to the cleavage furrow by targeted endosomal transport.^191^ This mechanism not only leads to a local area increase but also allows fast and localized delivery of specific lipids and regulatory proteins (reviewed in ref (192)). For reconstitution of cell division, various strategies can be followed to increase the membrane area (Figure 5, right). First, GUV membranes can be grown by external addition of small unilamellar vesicles (SUVs), which can be forced to fuse with the GUV using fusogenic peptides, DNA, or charge-based interactions.^193−196^ Second, lipid membranes can be grown by *in situ* synthesis of lipids from their precursors. Examples are non-enzymatic reactions from synthetic reactive precursors^197^ or enzyme-catalyzed biosynthesis using either purified proteins^198^ or *in vitro* transcription–translation.^199^ Although there is evidence that mammalian cells do not use area reservoirs, such as microvilli, to supply extra membrane area for division,^200,201^ this mechanism could be exploited to engineer division in synthetic cells. Asymmetries between the two leaflets of the bilayer generated by different means (see the preceding section) can be used to store excess area in membrane tubes and buds.^137,189,202,203^ Low forces suffice to access these reservoirs.^189,203^ To achieve synthetic cell division, it will be important to match the timing of membrane areal growth with the timing of actin-driven constriction. To achieve multiple cycles of division, it will moreover be important to build in a mechanism to maintain lipid homeostasis.

## 5 Challenges Ahead

In the past decades, our knowledge of cell division and its molecular actors has increased tremendously. To understand the physical mechanisms governing actomyosin-driven cell division, focus is put increasingly on bottom-up reconstitution experiments. Bulk and SLB experiments have helped us to understand the mechanics of active actomyosin networks in two and three dimensions. However, translating these insights to the process of cell division is not trivial. To summarize, we list here the critical challenges that need to be overcome before we can reconstitute a minimal version of actin-driven cell division.

First, we need to understand how actin network contraction is sustained to drive division all the way. This will require myosin activity working in concert with actin turnover. While activity and turnover have been studied to great extents individually, we still have minimal understanding of how they together govern actin network mechanics and contractility. Not only is this a challenging system to understand from a physical and biological perspective, but it is also difficult to recapitulate from an experimental perspective, as it involves a large number of components whose concentration and activity need to be tightly controlled. More *in vitro* work in this direction, in both two and three dimensions, will be essential to explore the parameter space.

Second, it remains elusive how the actomyosin network should be anchored onto the membrane in order to achieve membrane deformations. A multitude of anchoring strategies have been developed and investigated, but only minimally in combination with a deformable membrane. Combined with our limited understanding of cortex–membrane molecular organization *in vivo*, this might prove to be one of the most important challenges. Future studies need to focus on understanding the influence of linker density and strength as well as membrane composition and organization. In addition, the individual contributions of actin–membrane linkers and membrane-bound filament nucleators need to be delineated. After successful deformation, size stability between the two forming daughter cells, driven by the Laplace pressure, needs to be ensured.^204^

Third, attention must be paid to the supply of extra membrane area during constriction. Additional area can be present in membrane reservoirs, synthesized, or added by fusion of small vesicles. However, none of these approaches have to our knowledge been co-reconstituted with actin-driven contraction and resulting membrane deformation.

Fourth, to date there has been only a minimal body of work on contractile actomyosin networks in GUVs. Confining the system in GUVs requires that all of the components be encapsulated at the right concentrations and stoichiometric ratios while preserving functionality. Although there are numerous GUV formation techniques, they have been minimally characterized for their potential to encapsulate complex mixtures of biochemically active components. More work in this direction is crucial to perform controlled reconstitution in GUVs and also to be able to extrapolate findings from bulk and SLB experiments to vesicle systems.

Fifth, spatial and temporal control of the components and their activity is crucial. In the short term, some of the involved challenges may be bypassed by taking a semiautonomous approach to synthetic cell division. For example, optogenetics, external mechanical or chemical cues, and fusion-based delivery of components with small vesicles provide handles to control the system even after encapsulation of the components inside GUVs. However, if the goal is to create a synthetic cell that divides fully autonomously, reconstitution will be more complicated, requiring for example feedback loops, signaling molecules, and internal clocks.

As a concluding remark, we note that the most pressing challenges to achieve *in vitro* actin-driven cell division require integration of modules. Only when actomyosin studies meet membrane biophysics, when myosin motor activity is combined with actin turnover, and when protein biochemistry becomes integrated in GUV formation can we start thinking about reconstituting cell division. In the coming years, perspectives from experimental work, theoretical studies, and simulations need to be combined to guide future work with the ultimate goal of developing a full understanding of actin-driven synthetic cell division.
